# Clinical and Echocardiographic Outcomes After Aortic Valve Neocuspidization: Insights from a Large Multicentre Cohort

**DOI:** 10.1093/icvts/ivag175

**Published:** 2026-06-24

**Authors:** Kazuhiro Ueno, Tomoyuki Wada, Toshihiko Shibata, Kazuo Abe, Takashi Murakami, Shigeharu Sawa, Yoshito Inoue, Tamiyuki Obayashi, Kazushige Kanki, Shoichi Takahashi, Shinji Miyamoto

**Affiliations:** Department of Cardiovascular Surgery, Faculty of Medicine, Oita University, Yufu, Oita 879-5593, Japan; Department of Cardiovascular Surgery, Faculty of Medicine, Oita University, Yufu, Oita 879-5593, Japan; Department of Cardiovascular Surgery, Osaka City University, Osaka, Osaka 545-8586, Japan; Department of Cardiovascular Surgery, Yamagata Prefectural Central Hospital, Yamagata, Yamagata 990-2292, Japan; Department of Cardiovascular Surgery, Osaka City University, Osaka, Osaka 545-8586, Japan; Department of Cardiovascular Surgery, Ogikubo Hospital, Suginami, Tokyo 167-0035, Japan; Department of Cardiovascular Surgery, Tokyo Dental College Ichikawa General Hospital, Ichikawa, Chiba 272-8513, Japan; Department of Cardiovascular Surgery, Isesaki Municipal Hospital, Isesaki, Gunma 372-0817, Japan; Department of Cardiovascular Surgery, ShinSapporo Hospital of Cardiology, Sapporo, Hokkaido 004-0052, Japan; Department of Cardiovascular Surgery, Hoshi General Hospital, Koriyama, Fukushima 963-8501, Japan; Department of Cardiovascular Surgery, Faculty of Medicine, Oita University, Yufu, Oita 879-5593, Japan

**Keywords:** Ozaki procedure, aortic valve neocuspidization, AVNeo, reconstruction, autologous pericardium

## Abstract

**Objectives:**

Aortic valve neocuspidization (AVNeo) has gained widespread recognition in clinical practice; however, multicentre data describing its mid-term functional durability in real-world populations remain limited. This study aimed to evaluate the clinical and echocardiographic outcomes of AVNeo in a large multicentre cohort with extended follow-up.

**Methods:**

This retrospective multicentre study included 672 patients who underwent AVNeo at 16 institutions. The study population was characterized by advanced age, small body size, and a substantial burden of comorbidities. Mid-term outcomes included overall survival, freedom from reoperation, and longitudinal echocardiographic performance assessed from discharge through mid-term follow-up. Survival analyses were performed using the Kaplan-Meier method, and temporal echocardiographic changes were evaluated using linear mixed-effects models accounting for repeated measurements and centre-level clustering.

**Results:**

Early postoperative outcomes demonstrated acceptable procedural safety, with a 30-day mortality of 3.0%. Echocardiographic assessment showed functional optimization during the first postoperative year, while effective orifice area remained stable. Beyond 1 year, transvalvular gradients, peak velocity, valve area, and ventricular function remained stable up to 7 years after surgery. Estimated overall survival was 91.7% at 1 year and 79.5% at 5 years, while freedom from reoperation remained high at 99.4% at 1 year and 96.5% at 5 years.

**Conclusions:**

In this large multicentre real-world cohort, AVNeo demonstrated stable mid-term valve performance without evidence of clinically significant progressive deterioration. These findings support the durability and reproducibility of AVNeo in routine clinical practice.

## Graphical abstract

**Figure:**
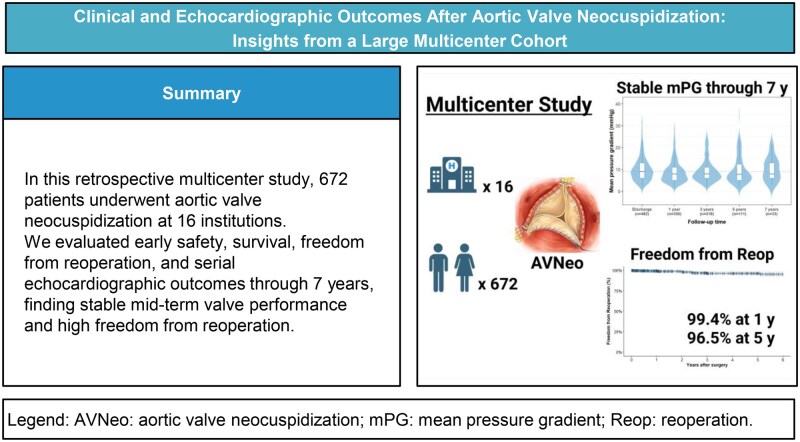

## INTRODUCTION

Surgical aortic valve replacement remains the standard treatment for severe aortic valve disease; however, prosthesis-related complications such as structural valve degeneration, prosthesis-patient mismatch, and the need for long-term anticoagulation continue to represent important clinical challenges, particularly in younger and more active patients.^1–3^ These limitations have driven ongoing interest in valve repair and reconstruction strategies that preserve native haemodynamics while avoiding prosthetic valve-related drawbacks.^4–6^

Aortic valve neocuspidization (AVNeo) using autologous pericardium has emerged as an alternative surgical technique that reconstructs anatomically tailored cusps, aiming to restore near-physiological valve function.^7^ Early and mid-term studies, including single-centre experiences and pioneering reports, have demonstrated favourable early outcomes and promising haemodynamic performance following AVNeo. Nevertheless, most available evidence is derived from relatively small cohorts or single-institution series, limiting the generalizability of these findings.^8–11^

In particular, comprehensive longitudinal echocardiographic assessment remains scarce. While survival and freedom from reoperation are essential clinical end-points, durable valve performance, reflected by transvalvular gradients, effective orifice area, ventricular function, and valvular regurgitation, is central to evaluating the true success of valve reconstruction techniques. Data on these parameters captured longitudinally in a large, real-world, multicentre population are currently limited.

Therefore, the present study aimed to evaluate the clinical and echocardiographic outcomes of AVNeo in a large multicentre cohort. By analysing real-world data with extended follow-up, we sought to assess the durability, functional performance, and clinical safety of AVNeo as applied in contemporary surgical practice.

## METHODS

### Review board approval

This multicentre observational study was approved by the institutional review board of the coordinating centre (approval number: 1209, April 2010), which served as the primary institution for study oversight and data management. Fifteen additional institutions participated as collaborating centres, and the study protocol was independently approved by the institutional review board of each participating institution. Written informed consent for the use and publication of clinical data and accompanying images was obtained from all patients. In addition, an opt-out option was provided through each institution’s website, allowing patients to withdraw consent at any time via online notification or direct contact.

### Patient selection

**Figure 1.**
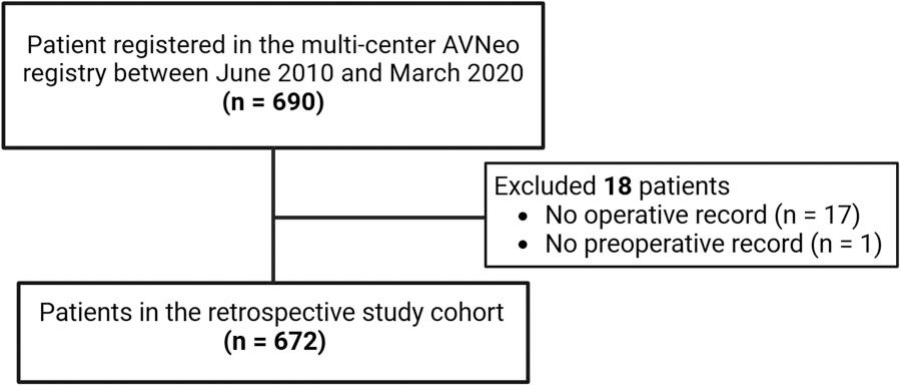
Flow Diagram of Patient Selection. Between June 2010 and March 2020, a total of 690 patients were registered in the multicentre AVNeo registry. After exclusion of patients without operative records (*n* = 17) or preoperative records (*n* = 1), 672 patients were included in the final retrospective study cohort. Abbreviation: AVNeo, aortic valve neocuspidization

Between June 2010 and March 2020, a total of 690 consecutive patients who underwent AVNeo using autologous or heterologous pericardium across 16 participating institutions were initially enrolled. Patient selection reflected real-world clinical practice at each institution; however, the principal indication for AVNeo was the presence of aortic valve disease requiring surgical aortic valve replacement, and surgical indications were determined in accordance with contemporary guidelines for valvular heart disease.^12^ Patients with a history of thoracic radiation therapy were excluded at all institutions because of concerns regarding pericardial quality. After exclusion of patients with missing essential operative or preoperative data, the final analytic cohort was defined as illustrated in **Figure 1**.

### Surgical technique

The AVNeo procedure was performed according to a standardized surgical protocol that has been previously described in detail,^13^ and all participating institutions adhered to this technique. To promote procedural standardization across participating institutions, surgeons were required to complete a dedicated workshop-based training program and obtain procedural certification before independently performing AVNeo. In addition, the initial AVNeo case at each participating institution was performed under direct proctoring by the developer of the AVNeo procedure. Briefly, autologous pericardium was harvested after removal of surrounding fat and redundant tissue, followed by treatment with a 0.6% buffered glutaraldehyde solution for 10 minutes. The pericardium was subsequently rinsed 3 times with sterile physiologic saline solution for 6 minutes per rinse.

All procedures were performed under cardiopulmonary bypass with cardioplegic arrest. After complete excision of the native aortic valve cusps, the intercommissural distances were measured using a standardized sizer system. Three new cusps were then trimmed from the treated pericardium using corresponding templates. Each cusp was sutured to the annulus with a continuous 4-0 monofilament suture, allowing for the creation of a natural 3-dimensional cusp configuration. Suturing was extended to the top of each commissure to achieve an extended coaptation zone at the level of the commissures, with additional commissural sutures placed to secure coaptation. Adequate cusp coaptation was confirmed by direct visualization prior to aortotomy closure.

In patients with Sievers type 0 bicuspid aortic valves, 2 additional commissures and annular attachment sites were created using the same sizing and suturing technique as for tricuspid valves, as previously described.^14^ In patients with aortic regurgitation or bicuspid or unicuspid valve morphology, circumferential reinforcement of the aorta at the commissural level using a 5-mm-wide felt strip was performed after aortic declamping to prevent future aortic dilatation and recurrent regurgitation.^14^

### Data collection and follow-up

Clinical, operative, and echocardiographic data were prospectively collected and managed using the Research Electronic Data Capture (REDCap) system.^15^ Patients were routinely followed at the outpatient clinics of their respective referral institutions. For the purposes of the present analysis, transthoracic echocardiographic data recorded in the registry at predefined follow-up categories, including prior to hospital discharge, and at 1, 3, 5, and 7 years postoperatively, were analysed. These data were collected as part of routine clinical practice at each participating centre, and the timing of assessments therefore reflects real-world follow-up rather than strictly standardized study intervals; no additional reassignment or rounding of time points was performed during the analysis. Echocardiographic assessment included transvalvular pressure gradients, effective orifice area, left ventricular systolic function, and the severity of aortic regurgitation, which was recorded using predefined registry categories (none, mild, mild-to-moderate, and severe). Before initiation of the registry, participating institutions attended a kickoff meeting to review the interpretation and grading criteria for echocardiographic parameters, including valvular regurgitation severity.

The primary outcomes of this study were mid-term clinical and functional outcomes, including overall survival, freedom from reoperation, and longitudinal echocardiographic performance. Early postoperative outcomes, including hospital mortality and perioperative complications, were assessed to evaluate procedural safety.

### Statistical analysis

Continuous variables are presented as median with interquartile range [IQR]. Categorical variables are expressed as counts and percentages. Longitudinal echocardiographic parameters were analysed using linear mixed-effects models with random intercepts for patients and centres to account for repeated measurements and clustering by participating centres. Overall survival and freedom from reoperation were estimated using the Kaplan-Meier method. Survival time was defined as the interval from surgery to death or last follow-up, with administrative censoring at 6 years given the limited number of patients at risk beyond this time point; patients alive at last follow-up or beyond 6 years were censored at that time point. Exploratory analyses were additionally performed to assess potential variation in baseline characteristics across centres and temporal changes over the study period, including descriptive comparisons and Cox proportional hazards models incorporating surgical era and clustering by centre. All analyses were conducted retrospectively using R Statistical Software (version 4.4.1). Survival analyses were performed using the survival package, and linear mixed-effects models were fitted using the lme4 and lmerTest packages.

## RESULTS

### Baseline patient characteristics

The baseline characteristics of the study population are summarized in **Table 1**. A total of 672 patients were included in the final analysis. The median age was 75.0 years (IQR, 68.0-80.0 years), and 324 patients (48%) were male. The median body mass index was 22.2 kg/m^2^ (IQR, 20.0-24.9). Preoperative comorbidities were relatively infrequent, with chronic obstructive pulmonary disease present in 2.8% of patients, peripheral vascular disease in 2.5%, and a history of stroke in 5.9%. Eighty patients (12%) were receiving maintenance haemodialysis. Infective endocarditis was the indication for surgery in 40 patients (5.9%). Most patients were symptomatic, with 82% classified as New York Heart Association (NYHA) functional class II or III. Regarding aortic valve pathology, aortic stenosis was the predominant lesion (55%), followed by aortic regurgitation (23%) and mixed disease (22%). Valve morphology was tricuspid in the majority of patients (80%), while bicuspid, unicuspid, and quadricuspid valves accounted for 17%, 1.2%, and 0.9% of cases, respectively. Ascending aortic enlargement was observed in 3.0% of patients.

**Table 1.** Baseline Patient Characteristics

	*N* = 672
Age (years)	75.0 (68.0, 80.0)
Sex (male)	324 (48%)
BMI (kg/m^2^)	22.2 (20.0, 24.9)
Body weight (kg)	54.3 (46.2, 61.0)
Chronic obstructive pulmonary disease	19 (2.8%)
Peripheral vascular disease	17 (2.5%)
History of stroke	40 (5.9%)
BNP (pg/mL)	167.7 (74.0, 440.0)
Creatinine (mg/dL)	0.9 (0.7, 1.1)
Haemodialysis	80 (12%)
Infective endocarditis	40 (5.9%)
NYHA class	
I	112 (17%)
II	419 (63%)
III	125 (19%)
IV	13 (1.9%)
Aortic valve disease	
Stenosis	368 (55%)
Regurgitation	157 (23%)
Mixed	147 (22%)
Aortic valve morphology	
Tricuspid	540 (80%)
Bicuspid	113 (17%)
Unicuspid	8 (1.2%)
Quadricuspid	6 (0.9%)
Ascending aortic enlargement	20 (3.0%)
Preoperative echocardiographic data	
LV ejection fraction (%)	64.1 (55.0, 72.0)
Peak pressure gradient (mmHg)	76.0 (50.0, 98.0)
Mean pressure gradient (mmHg)	45.0 (32.0, 59.0)
Peak velocity (Vmax, m/s)	4.2 (3.2, 4.9)
Aortic valve area (cm^2^)	0.7 (0.5, 0.8)
Aortic annulus diameter (mm)	20.1 (19.0, 22.6)
LV end-diastolic diameter (mm)	47.8 (42.0, 55.4)
LV end-systolic diameter (mm)	31.0 (25.1, 38.0)
Ascending aorta diameter (mm)	31.0 (27.0, 35.0)
Interventricular septum thickness (mm)	12.0 (10.7, 14.0)
LV posterior wall thickness (mm)	12.0 (10.0, 13.0)

*n* (%); median (Q1, Q3).

Abbreviations: BMI, body mass index; BNP, B-type natriuretic peptide; LV, left ventricular.

Preoperative echocardiography demonstrated preserved left ventricular systolic function, with a median ejection fraction of 64.1% (IQR, 55.0%-72.0%). Haemodynamic severity of aortic valve disease was substantial, with a median peak pressure gradient of 76.0 mmHg (IQR, 50.0-98.0), mean pressure gradient of 45.0 mmHg (IQR, 32.0-59.0), and aortic valve area of 0.7 cm^2^ (IQR, 0.5-0.8).

### Operative characteristics

Operative characteristics are summarized in **Table 2**. The vast majority of procedures were performed as primary surgeries (98%), with redo surgery accounting for only 2.2% of cases. The median aortic cross-clamp time was 135.0 minutes (IQR, 117.0-163.0), and the median cardiopulmonary bypass time was 183.0 minutes (IQR, 158.0-222.0). Conversion from AVNeo to conventional aortic valve replacement was required in 3 patients (0.4%). These conversions were primarily related to intraoperative technical considerations, including suboptimal valve competence due to sizing-related issues and technical challenges associated with extensive annular or valvular calcification. Concomitant cardiac procedures were frequently performed, reflecting the real-world surgical complexity of the cohort. These included coronary artery bypass grafting in 14% of patients, mitral valve procedures in 9.8%, tricuspid valve procedures in 5.4%, and ascending aortic replacement in 6.1%. Additional procedures included the Cox-Maze procedure (5.9%), aortic root reconstruction (2.8%), septal myectomy (1.3%), and other concomitant interventions (3.0%).

**Table 2.** Operative Characteristics

	*N* = 672
Primary/redo surgery	
Primary	655 (98%)
Redo	15 (2.2%)
Aortic cross-clamp time (minutes)	135.0 (117.0, 163.0)
Cardiopulmonary bypass time (minutes)	183.0 (158.0, 222.0)
Conversion to aortic valve replacement	3 (0.4%)
Concomitant procedures	
Coronary artery bypass grafting	96 (14%)
Mitral valve procedure	66 (9.8%)
Tricuspid valve procedure	36 (5.4%)
Aortic replacement	41 (6.1%)
Cox-Maze procedure	40 (5.9%)
Aortic root reconstruction	19 (2.8%)
Myectomy	9 (1.3%)
Others	20 (3.0%)
Leaflet size	
Left coronary cusp	25.0 (23.0, 29.0)
Right coronary cusp	27.0 (23.0, 29.0)
Non-coronary cusp	27.0 (23.0, 29.0)
Type of pericardium	
Autologous	658 (98%)
Heterologous (bovine)	12 (1.8%)

Median (Q1, Q3); *n* (%).

Regarding cusp reconstruction, the median leaflet sizes were 25.0 mm (IQR, 23.0-29.0) for the left coronary cusp, 27.0 mm (IQR, 23.0-29.0) for the right coronary cusp, and 27.0 mm (IQR, 23.0-29.0) for the non-coronary cusp. Autologous pericardium was used in the majority of cases (98%), while heterologous bovine pericardium was used in a small subset of patients (1.8%).

### Early postoperative outcomes

Early postoperative outcomes are summarized in **Table 3**. The median duration of intensive care unit stay was 4.0 days (IQR, 2.0-6.0), and the median postoperative hospital stay was 21.0 days (IQR, 15.0-30.0). The 30-day mortality was observed in 20 patients (3.0%). Postoperative complications included stroke in 30 patients (4.5%), complete atrioventricular block requiring intervention in 9 patients (1.3%), and cardiac tamponade in 11 patients (1.6%). Infectious complications were infrequent, with pneumonia occurring in 16 patients (2.4%) and deep sternal wound infection in 9 patients (1.3%).

**Table 3.** Early Postoperative Outcomes

	*N* = 672
ICU stay (days)	4.0 (2.0, 6.0)
Postoperative hospital stay (days)	21.0 (15.0, 30.0)
30-day mortality	20 (3.0%)
Complications	
Postoperative stroke	30 (4.5%)
Complete atrioventricular block	9 (1.3%)
Cardiac tamponade	11 (1.6%)
Pneumonia	16 (2.4%)
Deep sternal wound infection	9 (1.3%)

Median (Q1, Q3); *n* (%).

Abbreviation: ICU, intensive care unit.

### Echocardiographic outcomes

**Figure 2.**
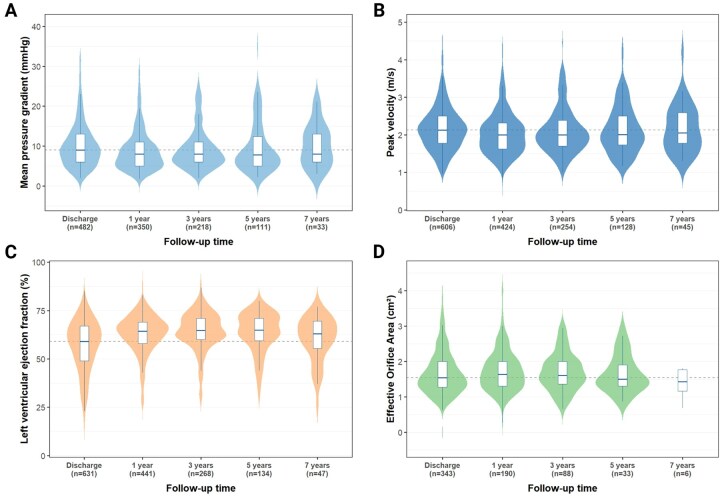
Longitudinal Echocardiographic Outcomes After Aortic Valve Neocuspidization. Violin plots depict the distribution of echocardiographic parameters at discharge and during follow-up at 1, 3, 5, and 7 years. (A) Mean transvalvular pressure gradient, (B) peak aortic jet velocity, (C) left ventricular ejection fraction, and (D) effective orifice area. Central boxes indicate median values with interquartile ranges

Longitudinal echocardiographic outcomes are illustrated in **Figure 2**. Marked haemodynamic improvement was observed early after surgery and was maintained throughout follow-up. Between discharge and 1-year follow-up, transvalvular haemodynamics improved. The median mean pressure gradient decreased from 9.0 mmHg (IQR, 6.0-13.0) at discharge to 8.0 mmHg (IQR, 5.0-11.0) at 1 year, a finding supported by linear mixed-effects modelling. Similarly, peak aortic jet velocity decreased from 2.12 m/s (IQR, 1.78-2.50) at discharge to 2.00 m/s (IQR, 1.63-2.32) at 1 year, also supported by linear mixed-effects modelling. Left ventricular systolic function improved during the first postoperative year, with median left ventricular ejection fraction increased from 59.0% (IQR, 49.1-67.0) at discharge to 64.3% (IQR, 58.0-69.0) at 1 year, a finding supported by linear mixed-effects modelling. In contrast, the effective orifice area remained stable between discharge and 1 year (1.54 cm^2^ [IQR, 1.27-2.00] vs 1.63 cm^2^ [IQR, 1.30-2.00]). Beyond 1 year, echocardiographic parameters demonstrated sustained valve performance without clear evidence of progressive deterioration. Mean pressure gradient, peak velocity, and left ventricular ejection fraction remained stable during follow-up, while effective orifice area was generally preserved. As shown in **Figure S1**, peak transvalvular pressure gradients followed a similar pattern, with early improvement and subsequent stability. These findings were confirmed in linear mixed-effects models, and the overall interpretation remained unchanged.

Longitudinal changes in aortic regurgitation grade are summarized in **Figure S2**. At discharge, none or mild aortic regurgitation was present in 94.5% of patients, whereas mild-to-moderate regurgitation was observed in 5.5%; severe regurgitation was not observed. The proportion of mild-to-moderate or greater aortic regurgitation was 16.8% at 1-year follow-up, 17.5% at 3 years, 19.3% at 5 years, and 20.8% at 7 years.

### Mid-term survival and freedom from reoperation

**Figure 3.**
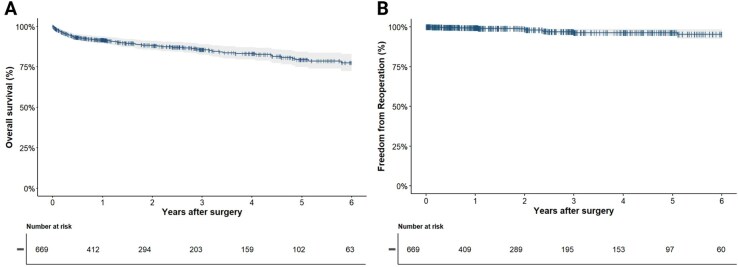
Mid-Term Clinical Outcomes After Aortic Valve Neocuspidization. Kaplan-Meier curves showing (A) overall survival and (B) freedom from reoperation following aortic valve neocuspidization. Shaded areas represent 95% confidence intervals. Numbers at risk are shown below each panel

Mid-term clinical outcomes are shown in **Figure 3**. Kaplan-Meier analysis demonstrated a gradual decline in overall survival during follow-up (**Figure 3A**), with estimated survival rates of 91.7% at 1 year, 85.7% at 3 years, 79.5% at 5 years, and 77.6% at 6 years. Among the 93 patients with available cause-of-death data, valve-related death was uncommon, occurring in 4 patients (4.3%), whereas most deaths were attributed to non-valve-related causes (71 patients, 76.3%); the cause of death was unknown in 18 patients (19.4%). Descriptive comparisons demonstrated heterogeneity in centre volume and baseline characteristics across participating centres. In exploratory univariable Cox analyses, several patient-related factors were associated with overall survival. The apparent association with surgical era was attenuated after accounting for centre-level clustering (**Table S1**), suggesting that this finding was driven by differences in centre participation over time rather than consistent temporal trends. Overall survival remained acceptable throughout the observation period.

Freedom from reoperation after AVNeo was high and remained stable over time (**Figure 3B**). Reoperations were required in 16 patients, including 10 who underwent valve re-repair and 6 who underwent valve replacement. The indications for reoperation included progression of aortic regurgitation in 4 patients, infective endocarditis in 4 patients, and progression of aortic stenosis in 3 patients, while 4 cases were attributed to other causes and 1 case had an unspecified indication. The estimated freedom from reoperation was 99.4% at 1 year, 96.5% at 3 and 5 years, and 95.5% at 6 years.

## DISCUSSION

This multicentre study demonstrates that AVNeo achieves durable functional and clinical outcomes in a real-world population characterized by advanced age, small body size, and substantial comorbidity burden. Aortic valve neocuspidization was associated with acceptable early postoperative safety, functional optimization during the first postoperative year, and stable valve performance without evidence of clinically significant progressive deterioration during mid-term follow-up. Despite baseline characteristics that differ markedly from those of many previously reported AVNeo cohorts, freedom from reoperation remained high, and longitudinal echocardiographic parameters remained preserved up to 7 years after surgery. These findings indicate that the benefits of AVNeo are reproducible beyond selected low-risk populations and specialized centres.

The patient population in this multicentre study differs substantially from many previously reported AVNeo cohorts, particularly those from European centres. The median age was 75 years, which is markedly higher than that reported in most European series (typically in the fifth or sixth decade),^9^^,^^16^^,^^17^ while body size was smaller, with a median body mass index of 22.2 kg/m^2^ compared with values around 26-29 kg/m^2^ in Western cohorts.^9^^,^^18^^,^^19^ In addition, patients receiving chronic haemodialysis accounted for 12% of the study population, a proportion comparable to Japanese reports but considerably higher than the 3%-6% reported in most studies.^11^^,^^20^ The relatively low proportion of redo procedures in this cohort likely reflects the predominance of first-time interventions at an advanced age, consistent with prior AVNeo reports.^8^ In addition, AVNeo is a technique that depends on the quality of the native pericardium, and therefore may be less frequently selected in patients with prior cardiac surgery or other conditions that could compromise pericardial integrity. This implicit selection in real-world practice may also contribute to the low proportion of redo cases observed in the present cohort. In this context, concomitant procedures were frequently required as part of comprehensive surgical management in this elderly population. The median aortic annular diameter was 20.1 mm, highlighting the frequent presence of small annuli in this cohort.^20^^,^^21^ These characteristics define a predominantly elderly, small-bodied, and high-risk population in whom prosthetic valve replacement is often limited by patient-prosthesis mismatch and accelerated calcification.^22^^,^^23^ Against this background, the favourable and durable outcomes observed in the present study emphasize the clinical relevance of AVNeo in patient populations that differ substantially from those previously reported.

Several prior studies have reported that valve haemodynamics after AVNeo continue to improve beyond the immediate postoperative period. For example, Iida et al^11^ reported a significant reduction in mean transvalvular pressure gradient from 11.7 mmHg at 1 week to 9.3 mmHg at mid-term follow-up, while Pirola et al^16^ demonstrated decreases in peak gradient and jet velocity between discharge and 3 months (from 16.2 to 10.9 mmHg and from 1.94 to 1.64 m/s, respectively). Consistent with these observations, the present multicentre cohort demonstrated further haemodynamic optimization during the first postoperative year, despite including an older and higher-risk population with smaller annular dimensions. Importantly, effective orifice area remained stable over time, a finding also reported by Krane et al,^9^ who observed unchanged valve area between discharge and 1 year. Together, these data support the concept that AVNeo is characterized by early postoperative functional maturation rather than immediate completion of valve performance, likely reflecting preserved annular dynamics and progressive adaptation of the reconstructed pericardial cusps. In addition, early reverse remodelling of the left ventricle following relief of aortic valve disease may also contribute to this progressive improvement, as suggested by recent longitudinal analyses of AVNeo demonstrating sustained ventricular remodelling over time.^24^

Sustained valve performance beyond the first postoperative year represents a defining feature of AVNeo and is consistently reported in prior studies. In the large series by Ozaki et al,^8^ the mean transvalvular pressure gradient remained stable up to 8 years of follow-up. Similarly, Benedetto et al^17^ reported stable mid-term gradients without evidence of progressive deterioration. In the present multicentre cohort, key echocardiographic parameters, including mean pressure gradient (approximately 8 mmHg), peak jet velocity (around 2.0 m/s), effective orifice area (about 1.5-1.6 cm^2^), and left ventricular ejection fraction, remained largely stable from 1 year through 7 years of follow-up. Although mild-to-moderate or greater aortic regurgitation was observed during follow-up, severe regurgitation remained rare, and the overall need for reoperation remained low. Importantly, these stable haemodynamic profiles were observed in an older and higher-risk population than those reported in most previous series, and remained robust in analyses accounting for repeated measurements and multicentre clustering. The number of patients at risk decreased at later follow-up time points, which may introduce variability in the distribution of echocardiographic measurements; therefore, mid-term stability should be interpreted in this context. Although overall survival declined gradually over time, exploratory analyses indicated that survival was primarily associated with patient-related factors, including age and comorbidity burden, whereas the apparent association with surgical era was attenuated after accounting for centre-level variation. This suggests that the observed decline in survival largely reflects the advanced risk profile of the cohort rather than valve-related factors. Together with the consistently high freedom from reoperation observed in this study, these findings reinforce prior evidence that AVNeo is characterized by durable mid-term valve function rather than time-dependent structural or functional deterioration.

The present findings have important clinical implications for the application of AVNeo in contemporary practice. The demonstration of stable valve haemodynamics maintained for up to 7 years, together with a high freedom from reoperation, suggests that AVNeo offers durable performance comparable to that reported in prior single-centre and European series, despite being applied in an older, smaller-bodied, and higher-risk population. These characteristics are particularly relevant in patients with small aortic annuli or chronic haemodialysis, in whom prosthetic valve replacement is frequently limited by patient-prosthesis mismatch and accelerated structural degeneration. By preserving native annular geometry and avoiding a prosthetic frame, AVNeo provides a physiologic reconstructive option that may expand surgical choices for such challenging patient subsets. Collectively, these data support the integration of AVNeo as a practical and reproducible surgical option in routine clinical decision-making, rather than a niche technique restricted to selected centres or patient populations.

Several limitations should be acknowledged. First, this study was a retrospective observational analysis and is therefore subject to potential selection bias and unmeasured confounding. Second, echocardiographic follow-up was incomplete at later time points, and longitudinal analyses were based on available data rather than repeated measurements in all patients, reflecting real-world clinical practice. In addition, echocardiographic assessments were performed at individual participating institutions without centralized adjudication, and therefore, inter-institutional variability in grading may have influenced longitudinal echocardiographic findings. Finally, the absence of a contemporaneous control group undergoing prosthetic aortic valve replacement precludes a direct comparative conclusion.

## CONCLUSION

In this large multicentre real-world cohort, AVNeo demonstrated stable mid-term valve performance without evidence of clinically significant progressive deterioration. Longitudinal echocardiographic assessment showed early postoperative functional maturation followed by sustained stability over extended follow-up. These findings indicate that AVNeo can achieve durable and reproducible outcomes when applied in routine clinical practice.

## Supplementary Material

ivag175_Supplementary_Data

## Data Availability

The data underlying this article will be shared on reasonable request to the corresponding author.
