# Supplementary material for: Clinical and Echocardiographic Outcomes After Aortic Valve Neocuspidization: Insights from a Large Multicentre Cohort
Source: Interdiscip Cardiovasc Thorac Surg. 2026 Jun 24;41(7):ivag175. doi: 10.1093/icvts/ivag175 (PMC13368825; doi:10.1093/icvts/ivag175)
Supplement: ivag175_Supplementary_Data [file ivag175_supplementary_data.zip › Supplementary Materials_r1_20260511.docx]

Supplementary Materials

**Supplementary Figure Legends**

**Supplementary Figure S1. Longitudinal changes in peak transvalvular pressure gradients after aortic valve neocuspidization.**

Violin plots illustrate the distribution of peak transvalvular pressure gradients at each follow-up time point. Central boxes represent median values with interquartile ranges. Peak gradients demonstrated early postoperative improvement followed by stable hemodynamic performance during follow-up, consistent with the findings for mean pressure gradient and peak velocity.

**Supplementary Figure S2. Longitudinal distribution of aortic regurgitation grade after aortic valve neocuspidization.**

Stacked bar plots demonstrate the distribution of aortic regurgitation severity at discharge and during follow-up at 1, 3, 5, and 7 years. Aortic regurgitation was categorized using predefined registry categories: none, mild, mild-to-moderate, and severe. Most patients demonstrated none or mild aortic regurgitation throughout follow-up, whereas severe regurgitation remained rare.

**Supplementary Table**

**Supplementary Table S1. Exploratory Cox proportional hazards analyses of baseline and structural factors associated with overall survival**

|  | **Univariable analysis** | | | **Center-clustered analysis** | | |
| --- | --- | --- | --- | --- | --- | --- |
|  | **HR** | **95% CI** | **P value** | **HR** | **95% CI** | **P value** |
| Age (per year) | 1.03 | 1.01–1.06 | 0.011 | 1.03 | 1.00–1.06 | 0.079 |
| Hemodialysis | 4.99 | 3.14–7.92 | <0.001 | 4.22 | 2.84–6.27 | <0.001 |
| Log BNP | 1.52 | 1.20–1.93 | <0.001 | 1.17 | 0.98–1.40 | 0.091 |
| NYHA class III–IV | 2.38 | 1.52–3.71 | <0.001 | 1.69 | 0.88–3.24 | 0.114 |
| Concomitant procedure | 1.65 | 1.07–2.54 | 0.024 | 0.92 | 0.58–1.46 | 0.718 |
| Surgical era (2015–2020 vs 2010–2014) | 2.26 | 1.36–3.76 | 0.002 | 2.05 | 0.85–4.91 | 0.109 |

Univariable Cox proportional hazards models were used to assess the association between baseline characteristics and overall survival. For center-adjusted analyses, Cox models with robust standard errors accounting for clustering by center were applied. Surgical era was defined as early (2010–2014) and late (2015–2020). BNP values were log-transformed due to skewed distribution. HR, hazard ratio; CI, confidence interval; BNP, B-type natriuretic peptide; NYHA, New York Heart Association.
