# Supplementary figures and images for: Clinical and Echocardiographic Outcomes After Aortic Valve Neocuspidization: Insights from a Large Multicentre Cohort
Source: Interdiscip Cardiovasc Thorac Surg. 2026 Jun 24;41(7):ivag175. doi: 10.1093/icvts/ivag175 (PMC13368825; doi:10.1093/icvts/ivag175)

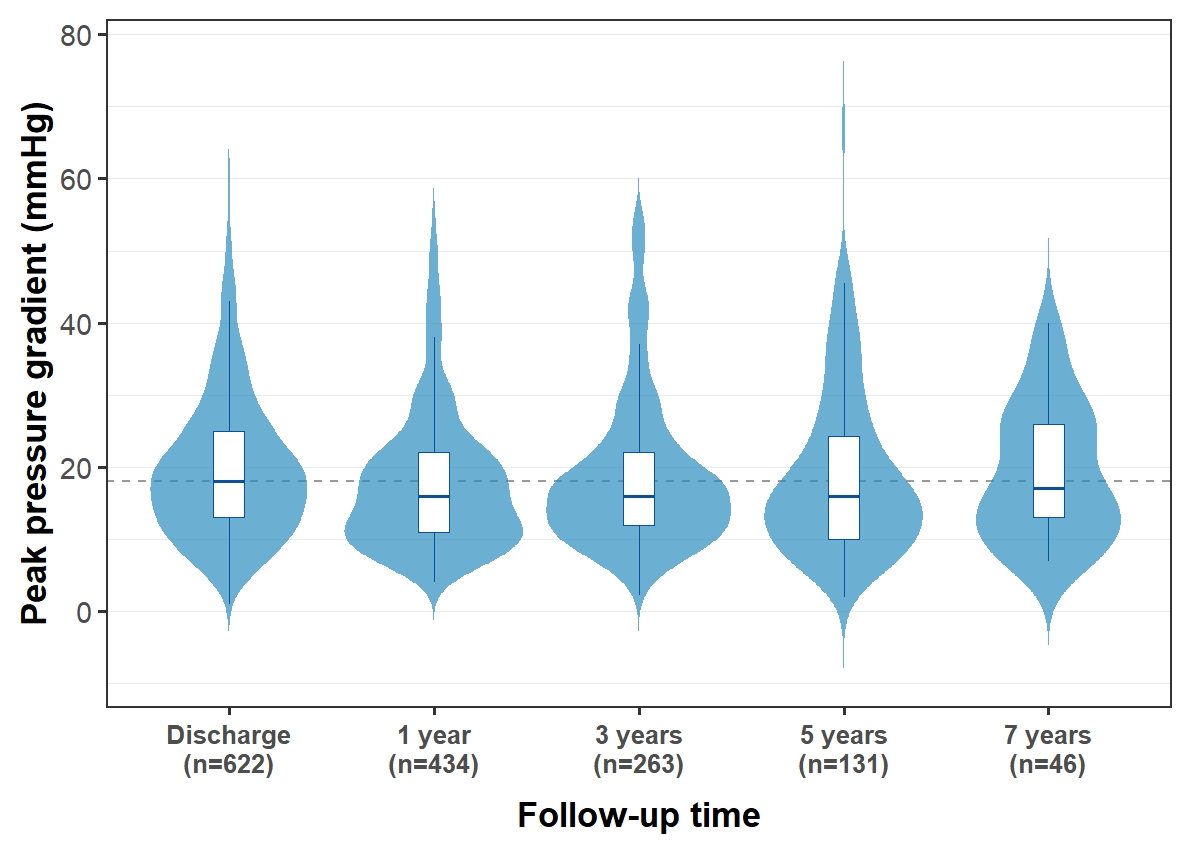

Supplement: ivag175_Supplementary_Data [file ivag175_supplementary_data.zip › Supplementary Figure S1.jpeg]

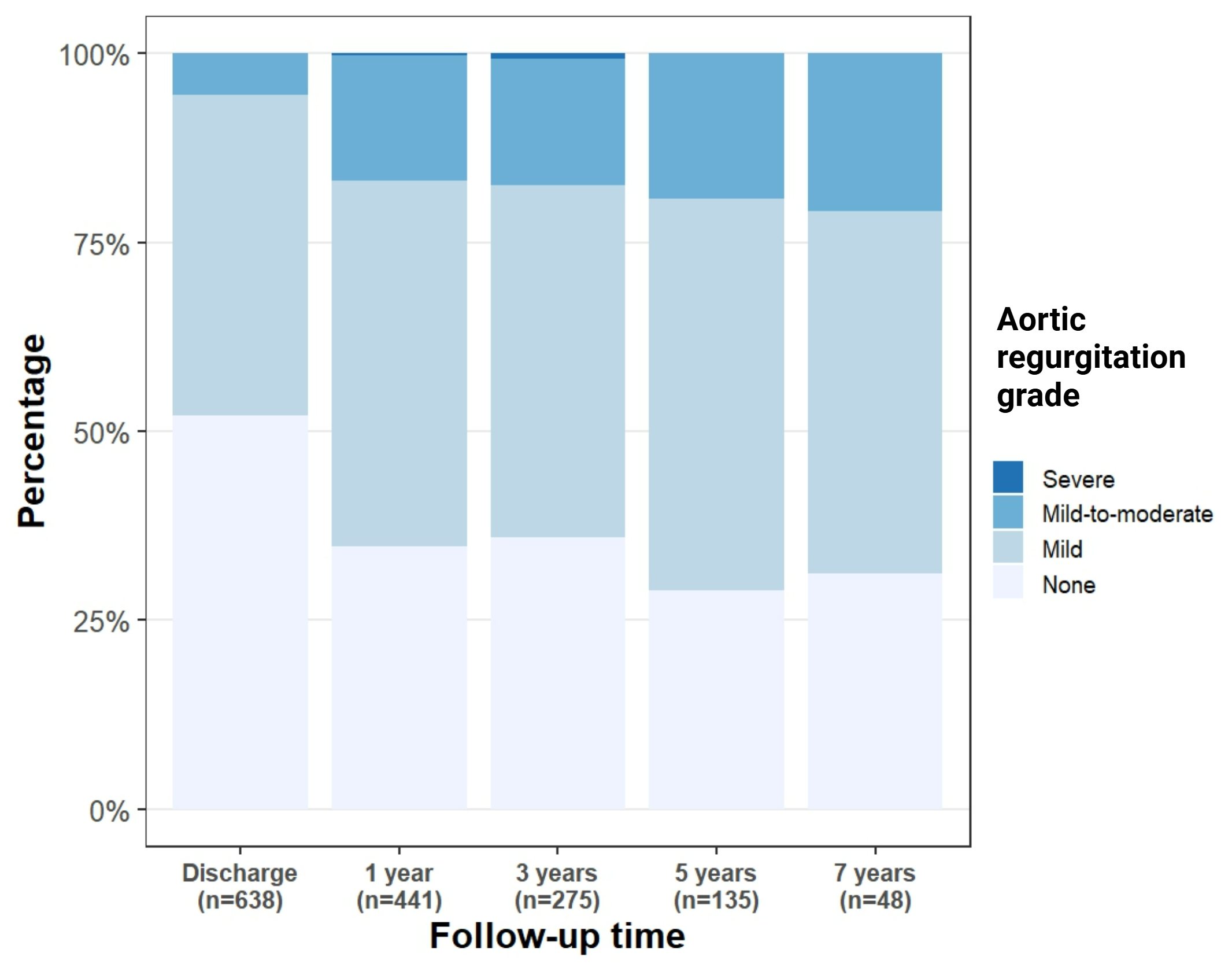

Supplement: ivag175_Supplementary_Data [file ivag175_supplementary_data.zip › Supplementary Figure S2.jpeg]
